# Current cancer driver variant predictors learn to recognize driver genes instead of functional variants

**DOI:** 10.1186/s12915-020-00930-0

**Published:** 2021-01-13

**Authors:** Daniele Raimondi, Antoine Passemiers, Piero Fariselli, Yves Moreau

**Affiliations:** 1 ESAT-STADIUS, KU Leuven, Leuven, 3001 Belgium; 2 Università di Torino, Torino, Italy, Torino, 10123 Italy

**Keywords:** Cancer driver variant prediction, Clever Hans effect, Bias in machine learning

## Abstract

**Background:**

Identifying variants that drive tumor progression (driver variants) and distinguishing these from variants that are a byproduct of the uncontrolled cell growth in cancer (passenger variants) is a crucial step for understanding tumorigenesis and precision oncology. Various bioinformatics methods have attempted to solve this complex task.

**Results:**

In this study, we investigate the assumptions on which these methods are based, showing that the different definitions of driver and passenger variants influence the difficulty of the prediction task. More importantly, we prove that the data sets have a construction bias which prevents the machine learning (ML) methods to actually learn variant-level functional effects, despite their excellent performance. This effect results from the fact that in these data sets, the driver variants map to a few driver genes, while the passenger variants spread across thousands of genes, and thus just learning to recognize driver genes provides almost perfect predictions.

**Conclusions:**

To mitigate this issue, we propose a novel data set that minimizes this bias by ensuring that all genes covered by the data contain both driver and passenger variants. As a result, we show that the tested predictors experience a significant drop in performance, which should not be considered as poorer modeling, but rather as correcting unwarranted optimism. Finally, we propose a weighting procedure to completely eliminate the gene effects on such predictions, thus precisely evaluating the ability of predictors to model the functional effects of single variants, and we show that indeed this task is still open.

**Supplementary Information:**

The online version contains supplementary material available at (10.1186/s12915-020-00930-0).

## Background

Cancer genomes present a very high mutation rate, but it is estimated that only a small fraction of the observed variants have a significant effect on tumor progression [1–3]. The variants identified by high-throughput DNA sequencing studies of cancer samples [4, 5] can be divided into “driver” variants, that provide a functional advantage to the cancer cell, and “passenger” variants, that do not favor tumor progression and are just a byproduct of the increased mutation rate and uncontrolled cell divisions typical of tumors [2, 5].

Reliable identification of driver mutations is crucial to improve our understanding of the molecular mechanisms underlying cancer [2, 6, 7] and to enable *precision oncology*, which aims at tailoring each patient’s treatment to the specific pattern of variants driving cancer progression [4, 7, 8]. Reliable functional characterization of variants found in cancer samples is thus of the utmost importance to fight this disease [7]. Currently, the validation of driver variants is done with in vivo or in vitro experiments [4, 7, 9], but these approaches cannot scale to the huge number of somatic mutations detected in cancer samples [10], and the most recent techniques allow only moderate-throughput testing [7].

To tackle this crucial challenge, in silico models aimed at the discrimination between driver and passenger variants have been developed [4, 5, 11]. However, determining the functional effects of variants is in general an extremely complex problem [4, 7, 12]. These computational approaches use various algorithmic solutions, that can be roughly divided into *supervised* machine learning (ML) methods [13, 14], *unsupervised* ML [15], and statistical approaches [6, 16].

Besides algorithmic differences, a crucial aspect of cancer driver prediction is that there is currently no consensus on which data should be used to evaluate their accuracy and for their development [7, 15]. Each tool is thus built using different data, and therefore the resulting methods tackle different *flavors* of the driver vs. passenger discrimination problem. For example, common definitions for passenger variants (negative samples) are (1) neutral germline variants, (2) deleterious (non-cancerous) germline variants, (3) both neutral and deleterious germline variants, and (4) non-recurrent variants from TCGA samples [17]. Similarly, two common definitions for driver variants (positive samples) are (1) experimentally verified cancer driver variants and (2) recurrent variants in TCGA samples. From these premises, it is clear that different combinations of driver and passenger definitions can be used to define positive and negative samples for the training and testing data sets for each predictor, which could influence dramatically (1) the patterns that the method is expected to learn and (2) the difficulty of the prediction task.

In this study, we benchmark 6 of the most widely used cancer driver variant predictors and 5 variant-effect predictors, which aim at discriminating deleterious from neutral germline variants, against 4 different definitions of passenger and driver variants. We show that the difficulty of the prediction challenge is affected by the design choices of the positive and negative samples in the data sets. Even more importantly, we show that the commonly adopted data set design choices could lead to learning a *Clever Hans* model [18], which exploits *confounders* in the data to provide correct predictions while following an invalid decision process (or at least different from what is assumed or claimed) [18]. In other words, a predictive model could deliver good predictions on a given data set while not solving the task it was intended for. More specifically, we show that following the vast majority of the data set building strategies, driver variants are *by definition* mapped on the few driver genes [3], while the vast majority of passenger variants are spread across all genes or they are even defined as variants *not mapped* on driver genes [10, 14].

These definitions allow models to *solve* the driver variant prediction task by just learning to discriminate driver genes from non-driver genes and use this *shortcut* to avoid the much more complex task of modeling the functional effects of each variant. To expose this *Clever Hans* behavior [18], we built the largest possible data set containing experimentally determined driver variants and passenger variants mapped on driver genes only, and we show that this constitutes the hardest task for the predictors. The gene-level information does not provide a useful *shortcut* to predict the single variants anymore, causing a 8-28 percentage-point drop in performance of the tested methods (in terms of area under the receiver-operating curve (AUC)). We thus propose this data set as validation set for the validation of the next generation of predictors, making it freely available as Additional file 1.

Moreover, we propose also a new validation procedure based on re-weighting the predictions errors on each gene, which is inversely proportional to the likelihood of the gene to host driver or passenger variants exclusively. Also, this approach is thought to be adopted during the validation of future methods, as it directly aims at evaluating the ability of predictors to compute predictions which are *truly* based on the functional effects of each variant.

## Results

### The notions of driver variant and driver gene are intertwined

Many cancer driver variant predictors have been developed so far, relying on different algorithmic solutions. Supervised methods need training and testing data sets, while unsupervised methods and statistical models need a testing data set to validate their performance. There is currently no agreement [7, 10, 11, 15] on a *gold standard* data set to train or validate these methods, and thus various design philosophies are usually followed by researchers, resulting each time in a different flavor of the driver vs passenger discrimination problem.

As shown in Table 1, common definitions for the negative samples (passenger variants) are (1) neutral germline variants (called NEUT in the rest of the article), (2) deleterious (non-cancerous) germline variants (called DEL), (3) both neutral and deleterious germline variants (ALL), (4) non-recurrent variants from TCGA samples (NONREC), and (5) synthetically generated passenger variants (SYNTH) [13]. Similarly, two common definitions for driver variants (positive samples) are (see also Table 1): (1) experimentally verified cancer driver variants (EXPDRV) and (2) recurrent variants in TCGA samples (REC).

**Table 1:** Summary of the different definitions of positive (driver variants) and negative (passenger variants) samples used in the construction of cancer driver variant predictors data sets

Negative samples		Positive samples	
Neutral germline variants	NEUT	Experimentally verified cancer drivers	EXPDRV
Deleterious germline variants	DEL	Recurrent variants in cancer samples	REC
Both DEL and NEUT	ALL		
Non-recurrent variants in cancer samples	NONREC		
Synthetically generated passenger mutation	SYNTH		

To show some examples of these definitions in the literature, we analyzed the state-of-the-art cancer driver variant predictors. The authors of FATHMM cancer [6] built the training set with positive samples defined as cancer-associated variants (germline and somatic) from Canprovar [19] and negative samples defined as neutral polymorphisms from Humsavar [20]. They tested the method using various data sets built as combinations of REC or EXPDRV versus NEUT, DEL, and ALL types of negative samples, providing an extensive validation of the performance. In CanDrA [14], the authors built the training set by defining positive samples as variants occurring in genes mutated in the cancer type under scrutiny that are observed in at least 3 primary tumor samples. The negative samples were defined as variants *not mapped* on cancer genes (taken from the COSMIC cancer census [21]) that are occurring only once in primary tumor samples. The positives and negatives in the testing data set are defined respectively as variants observed in at least 2 cancer samples and variants from hypermutated samples that are not mapped to cancer genes (taken from [21]).

In ChasmPlus [10], the positive samples are TCGA missense variants mapped to a curated set of 125 driver genes extracted from [22]. The negative class consists of the remaining missense variants on the same TCGA samples. The mutation frequency-based approach they used for this selection could nevertheless allow some passenger variants to be mapped on the 125 driver genes.

Also in ParsSNP [15] the authors tested their method on various combinations of REC and EXPDRV as positive samples and NEUT, ALL, and NONREC as negative samples.

Interestingly, in CHASM [13], the authors used as passenger mutations a set of synthetic variants. They randomly generated variants on genes which are known having mutations in at least four types of tumors. The strategy of sampling synthetic variants addresses the fact that the selective pressure that fixes germline variants in the population is likely very different from the pressure that fixes somatic mutations in tumors. The former allows the development of organisms to fit better the environment, while the latter only acts at the cell replication level. Thus, a benchmark that combines NEUT and DEL germline variants may still not fully represent the somatic passenger mutation landscape in tumors. Nevertheless, random sampling of realistic somatic passenger variants is not a trivial procedure either and depending on the strategy adopted also the synthetic variants might suffer from some deficiencies in their representativeness.

### Different definitions of driver and passenger variants influence the difficulty of the prediction task

In this study, we devised 3 data sets to determine how the data set design choices affects the difficulty of the prediction task. As a definition for positive samples, we used experimentally validated cancer driver variants extracted from CGI [23], which is an effort towards the integration and harmonization of experimentally determined cancer driver variants coming from different sources. We extracted 1991 variants from CGI and we refer to this data set as EXPDRV.

We coupled them with 3 definitions of negative samples, built by considering as passenger all the 63,525 germline variants (not related to cancer) in Humsavar [20] (we call this set ALL), only the 37,263 neutral variants in Humsavar (NEUT) and only the 28,252 deleterious variants in Humsavar. In this way, we thus built the EXPDRV vs. ALL, EXPDRV vs. NEUT and EXPDRV vs. DEL benchmark data sets.

Table 2 shows the performances of ParsSNP [15], CanDrA [14], CHASM [13], TransFic [16], ChasmPlus [10], FATHMM for cancer [6], DEOGEN2 [24], MetaSVM [25], CADD [26], Condel [27], and M-CAP [28] on these three benchmark data sets. We can see that the benchmarked methods have generally higher performances on the EXPDRV+NEUT data set, both in terms of Area Under the ROC curve (AUC) and Area Under the Precision Recall curve (AUPRC). This is probably because experimentally validated cancer driver variants have a very strong functional impact on the affected proteins [3], while the negative samples in the NEUT data set are germline variants with no or mild functional impact. This difference is further magnified by the fact that the majority of the samples in EXPDRV are somatic variants and thus are not subject to the same selective pressure of germline variants [3, 23]. The data set composed of driver variants from EXPDRV and a mixture of deleterious and neutral germline variants (EXPDRV+ALL) produces a harder prediction task, because of the higher functional impact of non-cancer-related germline deleterious variants (see Table 2), but the hardest prediction task in this benchmark is provided by the EXPDRV+DEL data set, because the non-driver germline deleterious variants used as negative samples have indeed a strong functional effect and are thus more difficult to distinguish from the functional effect of driver variants.

**Table 2:** Comparison of cancer driver variant predictors on the EXPDRV+NEUT and EXPDRV+ALL benchmark data sets

	EXPDRV+ALL		EXPDRV+NEUT		EXPDRV+DEL	
Method	AUC	AUPRC	AUC	AUPRC	AUC	AUPRC
ParsSNP	86.9	30.0	90.1	61.9	82.6	33.9
CanDrA v+	87.1	47.2	87.2	57.6	86.9	58.8
Chasm 3.1	91.1	54.5	94.0	73.7	87.3	58.8
CHASMplus	94.3	56.9	96.6	80.5	91.1	60.4
FATHMM cancer	91.0	39.1	93.2	67.3	87.7	43.6
TransFIC	68.0	6.0	80.6	22.0	51.0	7.9
Condel	64.3	4.5	83.9	24.3	38.5	5.6
DEOGEN2	68.8	4.6	91.3	43.7	38.9	5.2
CADD	73.7	6.4	88.5	26.5	54.0	7.9
M-CAP	54.0	4.9	83.1	37.9	38.9	5.4
MetaSVM	66.8	4.5	89.4	34.0	36.8	5.2
Dummy	98.1	46.6	98.7	67.9	97.4	59.7

Table 2 shows also that the methods specifically designed to discriminate driver from passenger variants (top section) have higher performance than variant-effect predictors (middle section), namely computational tools trained to distinguish between germline variants with neutral or deleterious effects (e.g., polymorphisms vs. rare variants causing genetic disorders). Even though some studies suggest that some of these predictors may be suitable to identify cancer driver variants [11], from Table 2, we can clearly see that they have relatively good performances only on the EXPDRV+NEUT, where there is a strong difference in the functional effect of the positive and negative variants. When the raw impact of the variant on the protein does not provide any signal for the discrimination (EXPDRV+DEL) data set, their AUC and AUPRC fall drastically, indicating that their reliability varies wildly as a function of the definition of positive and negative samples used to build the benchmark data sets.

### Cancer driver variants are unevenly distributed across human genes

From Table 2, we can see that changing the definition of passenger variant from neutral germline variants (with no or mild functional effect) to include deleterious germline variants (with more severe functional effect) causes a decrease of the prediction performances of the tested methods.

If we further analyze the construction strategy of these data sets, we notice an even more striking issue. In situations where the positive samples are defined as experimentally validated driver variants, these variants will be mapped on *cancer driver genes*, since the definition of “driver gene” is indeed based on the notion that it can host variants that either have activating effects (on oncogenes) or inactivating effects (on tumor suppressors) [3, 7, 29], effectively *driving* tumor progression. When the positive samples are defined as recurrent variants in cancer samples (e.g., from TCGA [17]), there is a risk of introducing too much noise through this labeling, since there is no definitive experimental evidence that all the recurrent variants contribute to tumor growth. Two strategies are commonly adopted to reduce this noise. The first is to define the positive samples as recurrent variants that map to curated sets of driver genes (as in [10]), and the second is to make sure that the *negative* samples do not map to known lists of cancer driver genes (as in [14]).

In all the cases mentioned so far, the adopted strategies end up building data sets in which all the positive samples map on a restricted subset of genes (driver genes), while the negative samples spread across the genome, with null or negligible intersection. While this appears to be a straightforward way to establish a robust *ground truth* labeling to develop predictive models, it provides, by construction, a *shortcut* that can allow the minimization of the classification error without considering the effects of the target variants at all, since it is sufficient to predict as positives all the variants mapping to driver genes to achieve an almost perfect AUC.

To show the extent of this behavior in the 3 data sets we used so far, we used conditional entropy *H*(label|gene)=*H*(label,gene)−*H*(gene) to quantify the amount of information needed to describe the driver/passenger status of the variants (*label*) given the “driver” status of the genes on which they are mapped. A situation in which *H*(label|gene)=0 indicates that the variable *gene* is sufficient to completely determine the variable *label*, while *H*(label|gene)/*H*(label)=1 indicates that the variables are independent. Computing *H*(label|gene)/*H*(label) then for the EXPDRV vs. ALL, NEUT, and DEL data sets used in Table 2, we respectively obtained 0.33, 0.24, 0.31 bits, indicating that knowing whether the gene hosting the variants is a driver gene conveys a significant part of the information needed to classify the variants on these data sets.

To provide empirical evidence for this phenomenon, we built a “dummy” predictor (see last row of Table 2) that implements the following strategy: for each variant, if it is mapped on a gene that contains at least one positive sample, it assigns it to the positive class; else, it assigns it to the negative class. Even though this Dummy predictor does not model in any way the functional effect of the variants, from Table 2, we can see that it outperforms all the other methods, reaching nearly perfect AUC on all data sets, outperforming the benchmarked methods. We can also see that the AUPRC scores obtained by Dummy, which are bounded by the ratio of driver and passenger variants on driver genes is similar to the best AUPRCs obtained by the other methods, suggesting that also *their* precision is impacted by the proportion of negative samples that are *unexpectedly* found on driver genes, since this situation is previously unseen in many of their training data sets.

### Forcing the predictors to discriminate between variants: a new benchmark data set

To test how these methods would perform in a situation in which no *shortcut* allows to bypass the real task of discriminating between the functional effects of variants, we built a specific benchmark data set. From the EXPDRV and ALL data sets, we considered only the genes with at least one positive and one negative sample mapped onto them. This data set, called DRGN (DRiver GeNes), contains 4093 variants mapping to 153 driver genes, and thus gene level information has a much more limited effect in discriminating between positive and negative samples.

Table 3 shows the AUC and the AUPRC of the same methods mentioned in Table 2 when they are tested on DRGN. The DRGN data set clearly provides the hardest prediction task proposed so far, since all the driver variant predictors show a significant drop in AUC between EXPDRV+ALL and DRGN (ParsSNP has the highest drop among with respect to Table 2). Interestingly, the AUCs of the variant-effect predictors are not as affected as in the EXPDRV+DEL case, because (1) they are not specifically trained to expect exclusively positive samples on driver genes and (2) the DRGN data set contains some neutral (low functional impact) variants among its negative samples, which these methods are able to pick up. The Dummy model, which was the better performing one in Table 2, is now producing random predictions, with an AUC of 50.0, because *all* the genes in DRGN are driver genes and thus this notion alone does not provide discriminatory signal anymore. The AUPRC of the Dummy model is still bounded to 44.2, which corresponds to the proportion of driver and passenger variants mapped on driver genes.

**Table 3:** Comparison of cancer driver variant predictors on the DRGN benchmark data sets. The last column shows the AUC drop with respect the EXPDRV+ALL data set in Table 2

Method	AUC	AUPRC	AUC drop
ParsSNP	59.4	52.0	27.5
CanDrA v+	66.2	64.7	20.9
Chasm 3.1	76.6	74.1	14.5
CHASMplus	72.3	69.8	22.0
FATHMM	69.1	65.9	21.9
TransFIC	59.6	53.0	8.4
Condel	55.9	51.7	8.4
DEOGEN2	57.2	48.7	11.6
CADD	63.6	55.5	10.1
M-CAP	47.4	45.9	6.6
MetaSVM	55.7	49.2	11.1
Dummy	50.0	44.2	48.1

**Fig. 1:**
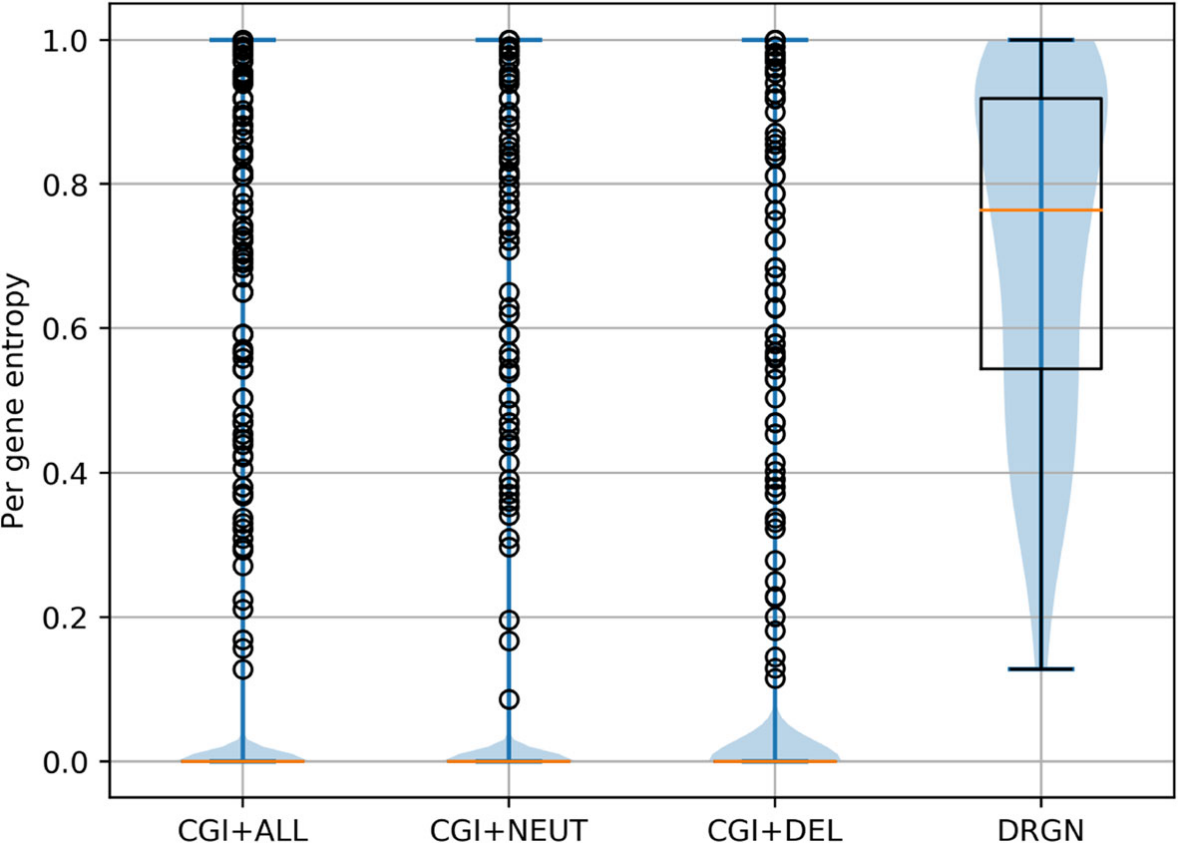
Per-gene entropy across the four data sets. The entropy distributions of all the data sets mentioned in Table 2 are heavily skewed towards 0, indicating that the genes in these data sets contain predominantly variants mapped on a single class, while the DRGN data set contains genes with significantly higher entropy

We further confirmed this result by computing the *H*(label|gene) conditional entropy of the DRGN data set, obtaining 0.99 bits, which is identical to *H*(label)=0.99 on the same data set. The fact that *H*(label|gene)=*H*(label) indicates that the driver/passenger status of the variants are independent from the driver gene status, meaning that knowing the status of the genes provides no information about the labels. This is also consistent with Fig. 1,which shows the distribution of the entropy computed on each gene across the 4 data sets. EXPDRV vs. ALL, NEUT, and DEL have distributions of gene entropy heavily skewed towards zero, meaning that the genes in these data sets contain variants predominantly from a single class, while the genes in the DRGN data set have a much higher entropy, indicating that they contain a much more balanced ratio of positive and negative samples. The Wilcoxon ranksums *p* values between the first three distributions in Fig. 1 and the DRGN distribution are always lower than 10^−90^ and the Cohen’s *d* effect sizes are respectively −7.9,−7.9,−4.7, indicating very large effects.

### All driver genes are equal, but some driver genes are more equal than others

The DRGN data set contains passenger and driver variants, which map to driver genes only, and thus does not contain a bias as obvious as EXPDRV vs. ALL, NEUT, or DEL. Additional file 2: Figure S1 shows the distribution of the log-odds of hosting driver vs. passenger variants for each gene in each data set, and we can see that DRGN has the most balanced distribution, with a mean close to 0 (perfect balance between positive and negative samples on the genes), while in the other data sets the genes have predominantly negative log-odds, indicating that the majority of the genes host predominantly passenger variants (also shown in Fig. 1).

**Fig. 2:**
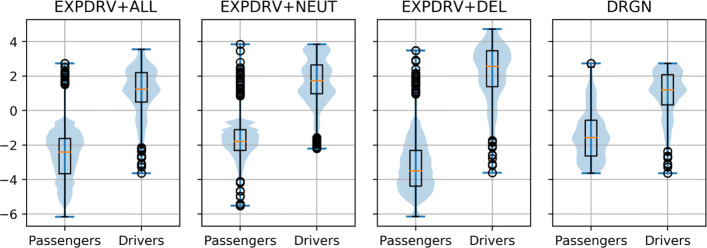
Gene log-odds of containing driver variants are still predictive. Plots showing the distributions obtained when, on the three data sets, each variant is represented by the log-odd of hosting positive samples of the genes to which they map

Once we deploy a nonlinear ML method on a data set, we rarely are in control of *what* it actually learns [18, 30], and thus, it is of the utmost importance to ensure that the data sets do not present hidden spurious patterns that we do not want to model or do not realize we are modeling. While DRGN mitigates the major driver gene bias present in the other data sets (see Fig. 1 and Additional file 2: Figure S1), this solution is not perfectly *safe* either. Figure 2 shows the distributions obtained when each variant *v* in the data sets is represented as the log-odds of hosting driver vs. passenger variants of the gene on which *v* is mapped. While the fact that driver variants are mapped on genes with higher tendency of hosting *only* driver variants is more pronounced on the EXPDRV+ALL and EXPDRV+NEUT data sets, we can see that also in DRGN driver and passenger variants are not always evenly mapped among genes, and thus, the log-odd the hosting gene can be used to identify, through its log-odd score, the likelihood of the class of each variant for prediction purposes. The Wilcoxon ranksums *p* values of the differences between the distributions are <10^−300^ in all cases and the Cohen’s *d* effect sizes are respectively −2.8,−3.7,−3.9,−1.8 standard deviations. To empirically show this possibility, we built the DummyLogOdd predictor, which simply assigns to each variant a prediction score equal to the log-odd of the hosting gene to contain positives vs negative samples. Even though DummyLogOdd does not model the functional impact of the variants, but only the ratio of positive and negative samples on each gene, it reaches an AUC of 88.7 and an AUPRC of 84.8 on DRGN.

Nevertheless, this hidden structure in the data is less dramatic than the one highlighted by the Dummy model in Table 2 on the EXPDRV vs. ALL, DEL, and NEUT data sets. First, not all genes are equal, and indeed they physiologically may have varying likelihoods to drive cancer growth upon mutation [4, 29]. Moreover, they are subject to different degrees of selective pressure and may be sensitive to different mutation patterns [31]. Second, this shows the importance of testing ML-based bioinformatics methods with a cross-validation (CV) that is *stratified* at the gene level, meaning that the cross-validation folds are not randomly split, but are specifically built to ensure that all the variants mapped on a gene *G*_*i*_ are in the same fold, thus ensuring that they are used *either* as training set or test set in each iteration of the CV [32]. Indeed, the log-odds used by the DummyLogOdd model are learned across the entire data set and applying a stratified CV would have prevented it to learn any log-odd for the genes in the fold used as testing, since they are not present in the folds used as training set. An even more stringent stratification would be selecting the genes in each fold in function of their sequence similarity, thus ensuring that genes with a sequence identity greater that 20 or 30% are assigned to the same fold, since above this *twilight* threshold some structural (and thus possibly functional) similarity is likely to be present [12, 32].

### A specific validation procedure for cancer driver variant predictors

As shown in Fig. 2, even while building a controlled data set to train or test driver variant predictors, it is not possible to remove all the gene-level patterns that could facilitate the prediction of the driver or passenger status of the variants, since part of these patterns indeed mirror existing biological mechanisms [4, 29].

To evaluate the extent to which driver variant predictors can model the functional effect of individual variants knowing that certain genes are more likely to host driver variants, we propose a novel scoring procedure that can be applied to any method and data set. Since the ease with which a predictor can predict the outcome *V* of a variant located on a gene *G* is negatively related to the entropy *H*(*G*), we weight the *cost* of erroneously predicted variants on each gene *G* in function of the likelihood of *G* to host predominantly negative or positive samples, effectively correcting the misclassification error for the entropy of each gene (in this case the entropy distributions are shown in Fig. 1). To do so, we upsampled variants belonging to the underrepresented class in each gene, thus obtaining a *true* estimation of how well a predictor can discriminate between the functional effects of variants disregarding the possible biases or gene-level patterns present in the data set.

Table 4 shows the AUC and AUPRC performances of 6 cancer driver predictors on the DRGN data set when we apply this weighting procedure designed to further remove gene-level information. All methods suffer from a further performance drop from the results on DRGN shown in Table 3, and an even more drastic drop with respect to the scores shown in Table 2, where no filtering was applied to the data sets. The best performing method is Chasm 3.1, but the low AUC score (64.5) indicates the real difficulty of discriminating between driver and passenger variants relying on the modeling of their functional impact alone, instead than exploiting contextual information, such as the genes on which they are hosted. Table 4 shows also that with this more stringent validation procedure, also the DummyLogOdd model produces random performance, because every gene now contains an equal number of positive and negative samples.

**Table 4:** Comparison of cancer driver variant predictors on the DRGN benchmark, with weighting of the predictions of the variants on each gene in function of the likelihood of *G*_*i*_ to host predominantly positive or negative samples. The last column shows the AUC drop with respect the EXPDRV+ALL data set in Table 2

Method	AUC	AUPRC	AUC drop
ParsSNP	52.9	52.3	34.0
CanDrA v+	51.7	52.1	35.4
Chasm 3.1	64.5	61.4	26.6
CHASMplus	59.2	57.9	35.1
TransFIC	57.5	54.8	10.5
FATHMM Cancer	57.6	54.7	33.4
DummyLogOdd	50.0	50.0	48.1

## Discussion

### Cancer driver variant prediction performance is overestimated

Prediction of mutations that can be characterized as passengers or drivers is critical in cancer research. Although researchers agree on the theoretical concept of passenger and driver variants, there are different operative definitions (see Table 1 for a summary), which lead to different prediction outcomes (see Table 2). A possible alternative data set for driver variants could have for example been [33], which provides a similar selection of driver genes and variants. Nevertheless, since the bias that we highlight in this study is rooted in the enrichment of positive samples on the few driver genes, any suitable selection of positive samples will show this effect, because the current notion of cancer driver variant is deeply connected with that of cancer driver genes, which consist of a tiny portion of the human proteome.

Another source of confusion is generated by the fact that current data sets allow predictors to mix up the effects observed at the gene level (driver vs. non-driver genes), with those found at the variant level (driver vs. passenger mutations), because driver variants map to driver genes only. This causes an overestimation of the prediction performance of cancer driver variant predictors, as shown by the huge drop in AUC and AUPRC when these methods are tested on data sets in which this bias is mitigated (see Tables 3 and 4).

**Fig. 3:**
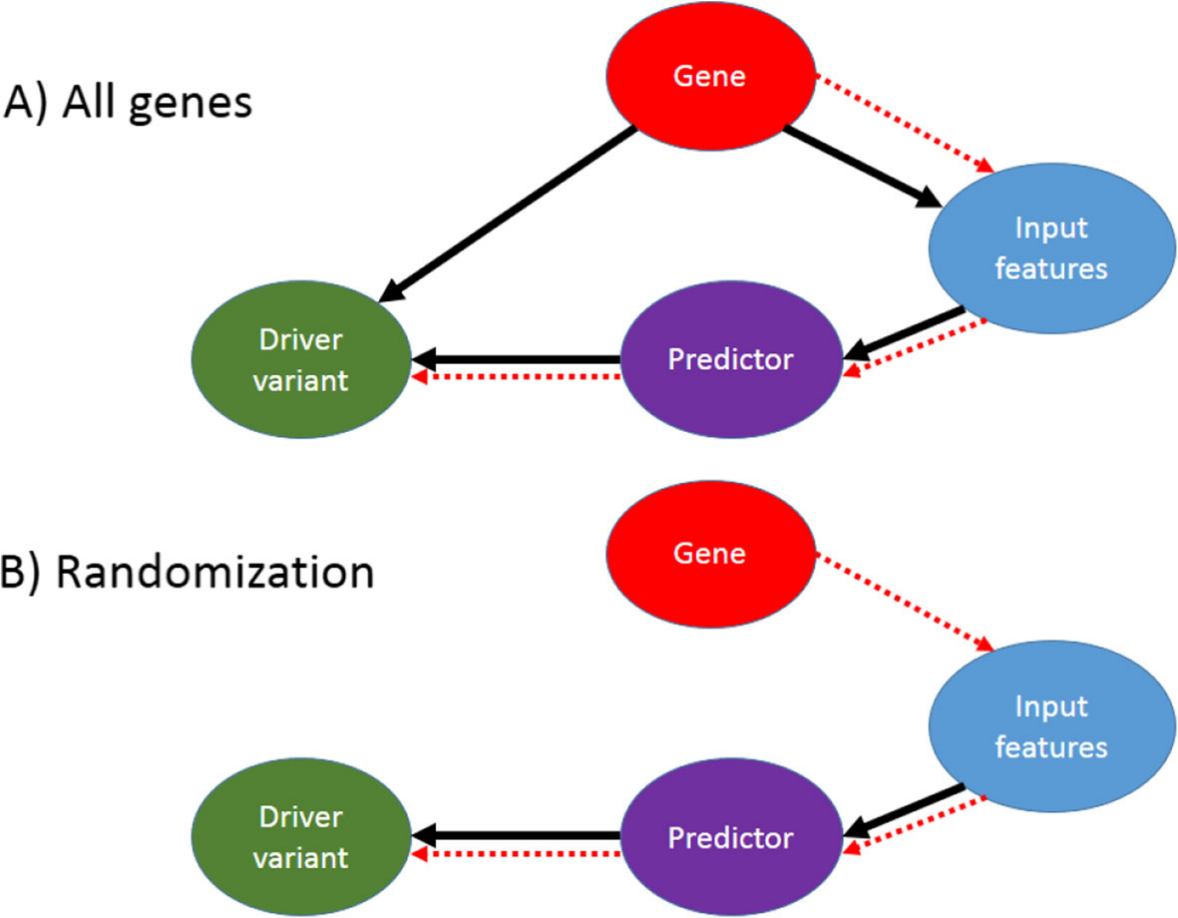
Dependency graph and prediction flux. The black lines represent conditional dependencies while the red dotted lines represent the information flow for a predictor

In Fig. 3, we visualize the conditional dependencies underlying this behavior. If there is a bias in the data set on the gene distributions (driver genes contain almost all driver variants and vice versa), the major predictor features come from the gene variable (Fig. 3a). However, we can remove the gene confounding effect using a randomization approach (Fig. 3b), as we did progressively in Tables 3 and 4, respectively by (1) building a more balanced data set and (2) by proposing a validation procedure that weighs the *cost* of each wrong prediction to mirror the gene-specific baseline difficulty of discriminating passenger and driver variants.

When we applied the re-weighting shown in Fig. 3b to the validation of cancer driver variant predictors, it produced a drastic drop in performances (see Table 4). This result indicates that, even though the published performances of cancer driver variant predictors kept increasing in the past years [6, 10, 11, 14, 15], this improvement was mostly apparent in and was likely the result of improvements in modeling the dependence of the inputs and the labels from the Gene variable (see Fig. 3a). The reassuring increase in performance of state-of the art methods thus masked the fact that their actual ability to truly model the molecular-level functional effects of each variant advanced at a much lower pace.

To reverse this trend, and thus ensuring that future cancer driver variant predictors will actually aim at modeling the effects of the variants—free from confounding effects—we propose two solutions. First, we provide a new test set composed of 4,093 variants over 153 genes in which every gene has at least one driver and one passenger mapped to it, called DRGN. Second, we propose a stringent validation procedure that implements the schema shown in Fig. 3b by using a re-weighting of the costs of prediction errors. Both approaches can thus be used for the unbiased validation of future predictors.

### The Clever Hans effect, or why prediction performance cannot be trusted without transparency in the prediction process

ML methods are valuable in countless tasks, including many major challenges in the biological sciences. Nevertheless, ML models are just functions parameterized by some trainable weights that are tuned to minimize a certain error produced by a loss function over a certain training data set. This means that, even in non pathologic bias-variance tradeoff conditions (e.g. optimal balance between under and over-fitting), the quality of the decision strategy learned by the model depends on (1) the data set on which it is trained and (2) the loss function used for the optimization. If the training set is truly representative of the general population of samples and the model is properly regularized, then the patterns learned by the model may be applicable to unseen cases, and the model will thus be able to *generalize* [18]. By contrast, even with a properly trained model, if confounders are present in the data used for modeling, the model can exploit such patterns to achieve apparently higher performance on the training and test set, while failing to actually model the real-world mechanisms that the researcher aims to characterize.

This problem is thus not one of overfitting, but rather a deeper issue related to the model robustness, which is not addressed by the conventional sequence-similarity based stratification approaches used to avoid information leakage during cross-validation [12, 24, 32]. Issues like this occur in various data sets and prediction tasks and they have recently gained attention in the ML community under the name of “Clever Hans” phenomena [18] from the analogous effect observed in psychology [18]. Various cases of Clever Hans predictors are known [34–36], with the notable cases of the debunked ML re-implementation of Cesare Lombroso’s approach to physiognomic criminology [37] that turned out to be detecting the presence of smiles instead of predicting criminal history and a “sexual orientation predictor” [38] that turned out to be a stereotype predictor [39].

Researchers developing predictors often use various means of biological contextualization [12, 24] to provide their ML with a more informed *worldview* on the task they need to solve, using gene- pathway- or tissue-level information as features describing each variant. On a well-balanced data set, this approach is indeed an effective way to improve the predictions, but if the data is biased or constructed in such a way that this contextual information is sufficient to *saturate* the prediction signal, this information could allow the ML to *bypass* the need to actually model the actual prediction task, which in this case is modeling the molecular-level functional effects of variants. While in carefully controlled settings using contextual information might add crucial pieces of information to the model, in other circumstances, this can lead to a Clever Hans predictor.

While researchers are driven to adopt more and more sophisticated ML methods to improve the state of the art, a comparable effort should be spent towards improving the quality of the training and testing data sets, thus analyzing the details for their structure to clean them from spurious correlations that could lead to learning Clever Hans models on them. Otherwise, ML methods may learn to exploit these correlations [34, 35], substantially *fooling* the researcher developing the model. The Clever Hans behavior can be quite difficult to detect, since at first glance it is indistinguishable from a successfully trained model. As argued in [18], a way out from this problem is to introduce as a standard procedure, besides the evaluation of the model performance, also efforts to *interpret* the model’s prediction strategy, thus investigating the meaningfulness of the correlations and patterns on which its predictions are based [18, 40].

Although nonlinear models have been considered hopelessly *black box* for a long time, methods that reach various degrees of detailed interpretation of their prediction strategy have been developed. Methods such as linear models, Random Forests [41], and Neural Networks [42, 43] are nowadays interpretable to a variable extent, and also model-independent approaches [18, 44] exist. Moreover, in the context of ML applied to biological sciences, investigating the decision strategy used by the model to classify the target samples has been shown valuable to improve our understanding of genetic disorders [41, 45] or molecular mechanisms that were unknowingly exploited by already existent *black box* approaches [46].

## Conclusion

Identifying driver variants is crucial to improve our understanding of cancer growth and for precision oncology. To help in this daunting task, various bioinformatics tools aimed at discriminating driver versus passenger variants have been developed, but although researchers agree on the need for this variant-level classification, there is no consensus for a *ground truth* labeling that could be used to define training and testing data sets. This leads to a variability of the definitions used for positive (driver) and negative (passenger) samples during the development of various predictors and benchmarks.

In this study, we investigated some of the main assumptions behind the construction of these data sets, showing that the different definitions used for the negative samples significantly influence the observed prediction performance. Moreover, we show that these data sets may lead, by construction, to learning a *Clever Hans* model if used as training sets. This is because the definitions of driver variants and driver genes are intertwined to the point that all the positive samples map to few driver genes, while passenger variants are widespread across thousands of genes. This bias allows any sufficiently complex model exploit this gene-level *shortcut* to classify driver and passenger variants by solving instead the simpler task of identifying the few driver genes, without needing to learn to model the functional effects of the single variants.

To overcome this issue, we provide a novel benchmark data set in which this bias is minimized, since it contains only driver genes on which *both* passenger and driver variants are mapped. Moreover, we propose also an even more stringent weighting procedure as additional validation, which completely removes the gene-level effects and allows to evaluate the extent to which ML methods can model the functional effects of variants.

Our analysis shows that the prediction of cancer driver variants consists of two complementary but distinct questions: Is the gene to which the variant map likely to be a driver gene? Is a given variant in a driver gene a driver or passenger variant? Considering predictive performance for the question “is this variant likely to be a driver variant or not?” is methodologically tricky because predicting that a variant in a known driver gene is a driver vs. passenger variant is a fundamentally different task from predicting that a variant is a driver variant in an uncharacterized gene.

## Methods

### Data sets

We retrieved 1990 variants that are demonstrated to drive tumor growth or predispose to cancer from the Cancer Genome Interpreter (CGI) [23], and we considered them as “positives” in our prediction. The data in CGI consist of a manually curated combination of the data available in the Database of Curated Mutations in cancer [47], ClinVar [48], and OncoKB [49]. We refer to this data set as EXPDRV.

From the November 2018 version of Humsavar [20], we retrieved deleterious and neutral variants. From this set, we removed all the variants (1) with “Unclassified” phenotypic annotation and (2) that are associated with cancer, both germline and somatic. Moreover, we removed from both Humsavar and EXPDRV data sets all the variants that were present in both with inconsistent functional annotation. After these filtering steps, we obtained 61,535 variants annotated with their deleterious or neutral phenotypic effect (26,260 of them are listed as deleterious, and 35,273 are neutral). We refer to this subset of Humsavar as ALL. From the ALL data set, we extract the subsets of neutral and deleterious variants, and we called them respectively NEUT and DEL.

We used these data sets to build 4 benchmark data sets for cancer driver variant predictors. In the first 3 data sets, we used the variants in EXPDRV as positive samples and we paired them with ALL, NEUT, and DEL variants as negative samples, obtaining the EXPDRV+ALL, EXPDRV+NEUT, and EXPDRV+DEL data sets. They contain respectively 63525, 37263, and 28252 variants.

The last data set is called DRGN and contains all the variants from EXPDRV and ALL that map to genes that are present in both data sets. In other words, the genes present in DRGN are the intersection of the genes in the EXPDRV and ALL data sets, and thus all these genes have at least one positive (driver) and one negative (passenger) variant mapped onto them. DRGN contains 4093 variants, with 1809 driver and 2284 passenger variants.

All the data sets are available as Additional file 1.

### Prediction methods

We used our 4 data sets to benchmark the most widely used cancer driver variant predictors. We retrieved CHASM 3.1 [13] predictions from the CRAVAT [50] web server, using the “Other” type of cancer. We installed locally CanDrA Plus [14] (http://bioinformatics.mdanderson.org/main/CanDrA) and used the “cancer-in-general” model. We obtained TransFIC [16] predictions from (http://bbglab.irbbarcelona.org/transfic/home). We retrieved CHASMplus [10] predictions by locally installing OpenCRAVAT [51]. We computed FATHMM-cancer [6] predictions from their web server (http://fathmm.biocompute.org.uk/cancer.html). We installed and ran locally ParsSNP [15]. We retrieved the predictions of CADD [26], DEOGEN2 [24], M-CAP [28], and MetaSVM [25] from dbNSFP [52].

### Implementation details

We computed the per-gene entropy *H*(*gene*) with the formula $H(gene) = -{\sum \nolimits }_{i = 0}^{i< n}P(x_{i}|gene) \log _{2} P(x_{i}|gene)$, where *n*=2 and *P*(*x*_*i*_|*gene*) corresponds to the probability of the gene to host negative (0) and positive (1) labeled variants.

The predictions of the Dummy model used in Table 2 have been computed by simply assigning 1 to each variant *v* if the gene on which *v* is mapped contains at least one positive variant (driver variant), and 0 otherwise.

The predictions of the DummyLogOdd model used in Table 4 have been computed by assigning to each variant *v* the log-odd of the gene on which *v* is mapped to host positive versus negative samples (driver vs passenger variants).

The procedure we propose in order to ensure a complete removal of the driver gene-based bias aims at re-weighting the cost of a wrong prediction in function of the entropy of the gene on which each variant is mapped, since variants on genes with high entropy are harder to predict. To do so, within each gene, we upsampled the variants belonging to the underrepresented class, artificially ensuring a per-gene log-odd of hosting driver vs passenger variants equal to 0. This procedure is general and can be performed on any data set, provided that the genes contain at least one positive and negative sample.

### Evaluation procedure

We evaluated the performances of the methods by using the area under the ROC curve (AUC) and the area under the precision recall curve (AUPRC) computed with the scikit-learn library [53]. We based our study on these two scores because they are independent from the threshold used to binarize the predictions into positive and negative classes, which may be a strong determinant for the evaluation of metrics, such as Matthews correlation coefficient (MCC), sensitivity, specificity, and precision. We thus preferred to remain agnostic about the optimal threshold that should be used to binarize their predictions and focus on evaluating their ability to consistently assign higher values to positive samples and vice versa.

## Supplementary Information

**Additional file 1** Zip file containing the datasets used as benchmark in this paper.

**Additional file 2** **Figure S1:** Plot showing the distributions of the per-gene log-odds of containing driver vs passenger variants over the four datasets. **Table S1:** Table showing the percentages of the missing predictions for each cancer driver predictor on each dataset. **Section S1:** details of the entropy computations.

## Data Availability

All the data used in this paper are publicly available as Additional file 1.
