# Supplementary material for: Current cancer driver variant predictors learn to recognize driver genes instead of functional variants
Source: BMC Biol. 2021 Jan 13;19:3. doi: 10.1186/s12915-020-00930-0 (PMC7807764; doi:10.1186/s12915-020-00930-0)
Supplement: Supplementary file 2 — Additional file 2 Figure S1: Plot showing the distributions of the per-gene log-odds of containing driver vs passenger variants over the four datasets. Table S1: Table showing the percentages of the missing predictions for each cancer driver predictor on each dataset. Section S1: details of the entropy computations. [file 12915_2020_930_MOESM2_ESM.pdf]

Supplementary Material

Figures

Figure S1: Plots showing the distributions of the per-gene log-odds of containing driver vs passenger variants over the four datasets. We can see that DRGN has the most balanced distribution. The Wilcoxon ranksums p-value of the differences between the first three distributions and the DRGN are respectively $6.7 \times 10^{-24}, 2.8 \times 10^{-19}, 9.0 \times 10^{-25}$ and the corresponding Cohen's d effect sizes are $-1.3, -1.4, -0.9$ standard deviations.

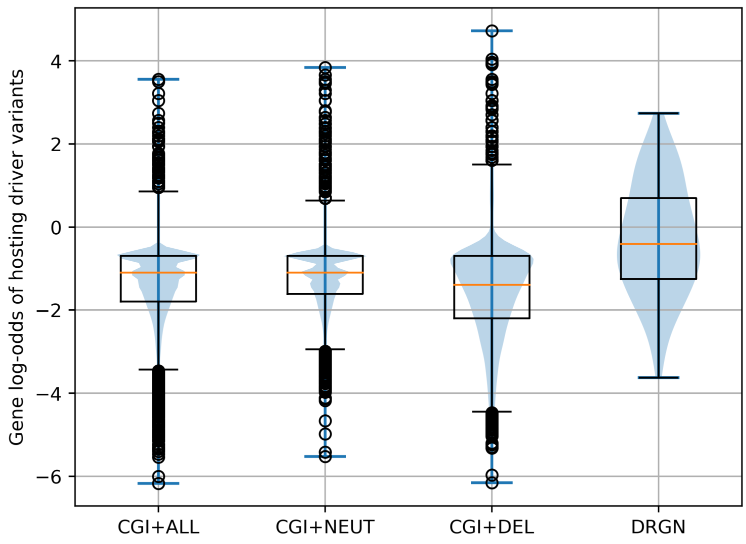

Tables

Table S1: Table showing the percentages of the missing predictions for each cancer driver predictor on each dataset. All the predictors except FATHMM have a negligible number of missing predictions.

Method	EXPR+ALL	EXPR+DEL	EXPR+NEUT	DRGN
ParsSNP	0.0	0.0	0.0	0.0
CanDrA v+	1.0	0.3	1.5	0.0
Chasm 3.1	0.2	0.1	0.3	0.0
CHASMplus	0.6	0.2	0.9	0.0
FATHMM	17.7	21.4	15.2	25.3
TransFIC	1.4	0.4	2.2	0.0

S1 Entropy computations

Entropy is computed as:

$$H(X) = -\sum_{i=1}^{n} p(X = x_i) \log_2 p(X = x_i)$$

The per-gene entropy for gene g is computed as:

$$H(X) = -\sum_{i \in g} p(X = x_i) \log_2 p(X = x_i)$$

The conditional entropy is computed as:

$$H(X|G) = -\sum_{j=1}^{m} \sum_{i=1}^{n} p(X = x_i, G = g_j) \log_2 \frac{p(G = g_j)}{p(X = x_i, G = g_j)}$$

where x_i is the class of variant i and g_j is the driver status of gene j.
